# Quantitative assessment of phased array coils with different numbers of receiving channels in terms of signal-to-noise ratio and spatial noise variation in magnetic resonance imaging

**DOI:** 10.1371/journal.pone.0219407

**Published:** 2019-07-05

**Authors:** Kyoung-Nam Kim, Daniel Hernandez, Jeung-Hoon Seo, Young Noh, Yeji Han, Yeun Chul Ryu, Jun-Young Chung

**Affiliations:** 1 Department of Biomedical Engineering, Gachon University, Incheon, Korea; 2 Neuroscience Research Institute, Gachon University, Incheon, Korea; 3 Department of Neurology, Gachon University Gil Medical Center, Incheon, Korea; 4 Department of Neuroscience, College of Medicine, Gachon University, Incheon, Korea; Wayne State University, UNITED STATES

## Abstract

The neuroimaging of humans using 7T magnetic resonance imaging (MRI) has been conducted using phased array (PA) coils with different numbers of receiving channels. PA coils with a high number of channels may offer parallel imaging (PI) with a high reduction (R)-factor, which is enabled via under-sampling and coil geometry (*g*) factor, increasing the radiofrequency signal sensitivity provided by a small coil. The goals of this study were to assess and validate the coil performance of PA coils with different numbers of receiver (Rx)-channels in and to propose the coil selection guidelines by visualizing 7T brain images. The combined magnetic flux density (||B_1_||) distributions of four configurations of PA coils—4-, 8-, 12-, and 16-channel Rx-only mode under the local transmit (Tx) mode of birdcage coils—were evaluated using electromagnetic (EM) calculations. These four configurations of PA coils and a local Tx coil were designed and built for a 7T MRI experiment. For 7T brain imaging experiments, all PA coils with (*w*/) and without (*w*/*o*) R-factors were compared in terms of signal-to-noise ratio (SNR) and spatial noise variation (SNV). EM simulation results clearly demonstrated that PA coils with a high number of Rx channels showed more homogeneously distributed ||B_1_|| fields than a PA coils with a low number of Rx coils. The results of this study demonstrate that a collection of smaller surface coils can contribute to high RF signal sensitivity in terms of the anatomical coverage of the brain and may facilitate PI. With further improvement in coil technology, researchers and clinicians will be provided with PA coils with different numbers of channels, which can ensure the optimum SNR and PI benefits for 7T brain MR imaging.

## Introduction

Ultra-high-field (UHF, ≥7T) magnetic resonance imaging (MRI) is being continuously developed to obtain high-quality neuroimages. Increase in signal to noise and susceptibility induced contrast at higher field strengths may be useful for imaging techniques that require a high signal-to-noise (SNR) and contrast-to-noise (CNR) ratio, such as functional imaging techniques (fMRI) and MR spectroscopy, as well as high resolution anatomical imaging. For this purpose, the MR scanner should provide a high signal-to-noise ratio (SNR) while maintaining the acquisition time short. To meet these requirements, the utilization of high-magnetic field (||B_0_||) strength is essential. S/N can be improved by the application of stronger ||B_0_||-fields due to the property of MR signals that the acquired MR signals are proportional to the number of hydrogen (^1^H) protons entering the resonance state, which is in turn proportional to ||B_0_||-field strength [1, 2]. Moreover, in a UHF environment small coils are less influenced by noise. Therefore, the coupling of a small-surface radiofrequency (RF) coil, which is the multiple element of a phased array (PA) coil, is used to enhance the overall signal sensitivity and extend the field of view (FOV) [1, 3]. Notably, the SNR for *n* number of well-decoupled receiver coils can be increased √n-times the S/N for a single-coil element [4, 5]. In addition, the use of multiple elements in PA coils allows parallel imaging (PI) associated with inherent spatial sensitivity, which is used to increase acquisition speed. The characteristics of PA coils of enabling the acquisition of unique images with different coil elements has been used for PI reconstruction techniques, such as Sensitivity Encoding (SENSE) [6], Simultaneous acquisition of spatial harmonics (SMASH) [7], generalized auto-calibrating partial parallel acquisition (GRAPPA) [8] and array coil spatial sensitivity encoding (ASSET) [9]. The performance of PI techniques is dependent upon ||B_0_||-field strength [10]; for example, in theory a high field strength can provide reduction (R)-factors ranging from 4 to 6. In practice, the R-factor depends upon the capability of PA coils to decouple each coil element. Array coils with multiple receivers, with up to 32 channels, have been developed [11] in 7T MRI systems. The use of PI is important when the scan time is long, which requires the patients to hold their breath and avoid motion. The drawback of increasing the magnetic ||B_0_||-field strength is that the ||B_1_||-field distribution becomes inhomogeneous. The ||B_1_||-field inhomogeneity is due to a relative reduction in the RF wavelength inside the imaging object. Therefore, RF coils need to be carefully developed to achieve uniform ||B_1_||-fields in the transmission (Tx) and reception (Rx) modes. As a PA coil consists of *n* number of coil elements, the mutual inductance or coupling between each element needs to be carefully optimized. Methods of reducing the mutual inductance coupling, include geometrical decoupling such as overlapping [12], non-overlapping, or shared coil geometry, in addition to capacitive, inductive [13], and transformer decoupling [14]. In this study, we aimed to determine the optimum number of channels in a PA coil that would yield a better SNR at different R-factors when used for obtaining PI. Four configurations of PA coils comprising 4, 8, 12, and 16 channels under the additional local Tx-only birdcage (BC) coil are proposed. The coils were designed to operate at a frequency of 300MHz, which corresponds to a frequency of 7T MRI systems. We obtained ||B_1_||-field distributions from each coil using electromagnetic (EM) field simulations. The PA coils were designed and built to minimize the coupling between the coils using geometry overlapping. Bench measurements were performed to determine the decoupling level between the coil elements. Finally, the coils were tested by performing *in vivo* brain experiments with different R-factors in terms of SNR and spatial noise variation (SNV).

## Materials and methods

### ||B_1_||-field distribution of n-channel PA head coils

**Fig 1:**
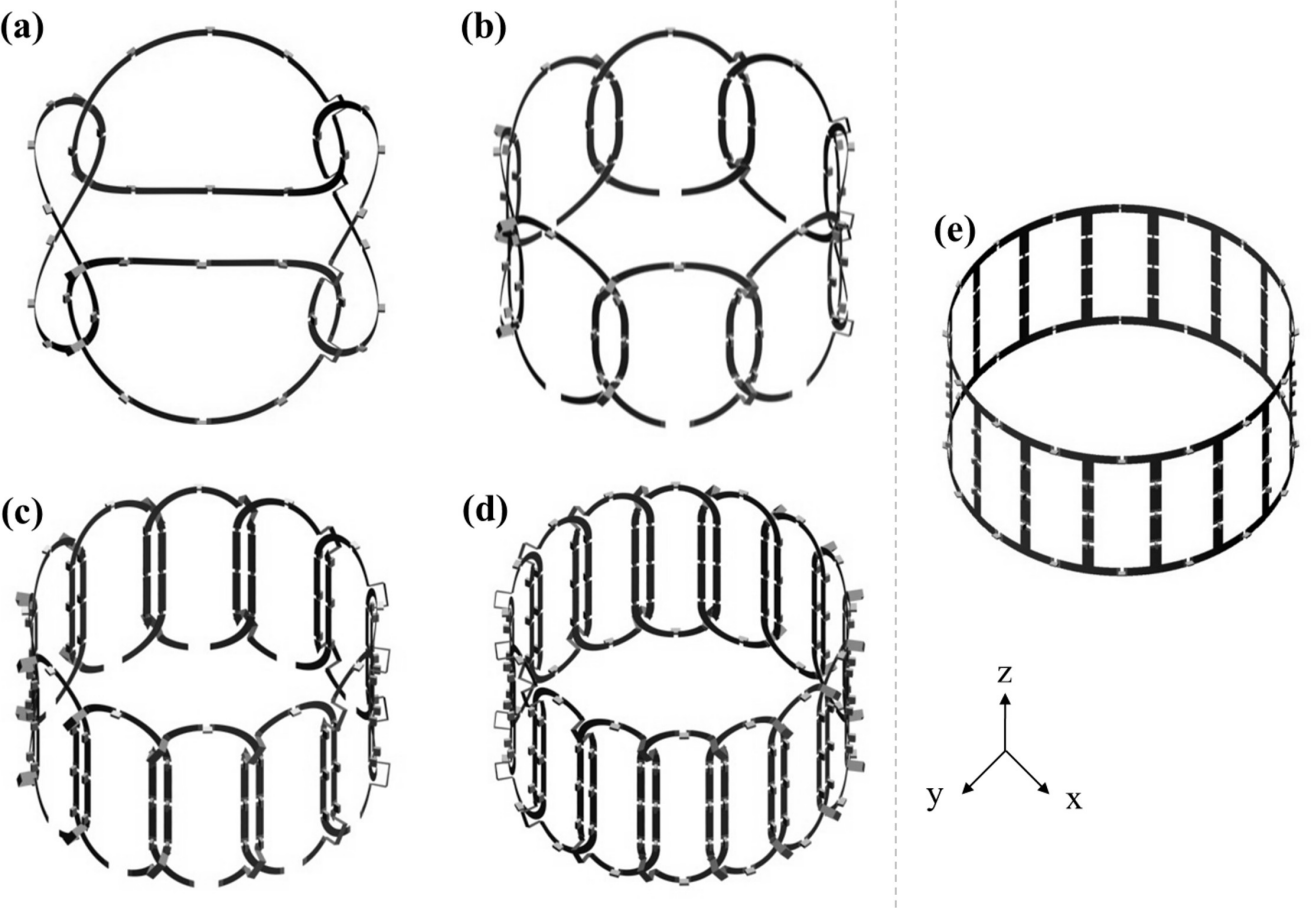
(a–d) Modeling of the four PA coils of 4, 8, 12, and 16 channels and (e) band-pass filter BC coil.

Numerical methods were applied to evaluate and design the coil geometry before the construction of RF coil. EM simulations were performed for the four different PA coils and a BC coil using the finite-difference time-domain (FDTD) simulation software Sim4Life [Zurich Med Tech AG (ZMT)]. For the quantitative assessment of the ||B_1_||-field distribution, simulations were performed by discretizing into a 764×764×381 matrix in the *x*-, *y*-, and *z*-directions. The RF excitation frequency for the FDTD simulations was 300MHz, which corresponds to the Larmor frequency of 7T MRI systems. The four PA coils comprising 4, 8, 12, and 16 channels were modeled around a cylindrical geometry, with a height of 220mm and a diameter of 135 mm, as shown in Fig 1(A)–1(D). Each coil element had a height of 135mm for all coils, and width of 202.5, 111.3, 72.5, and 58.1mm for 4, 8, 12, and 16 channel PA coils, respectively. The PA coils were designed to produce geometric decoupling to minimize the mutual inductance between the channels [5,12] by overlapping with the adjacent coil elements. The overlapping distance between the coil elements was 25, 20, 10, and 8 mm for the 4-, 8-, 12-, and 16-channel PA coils, respectively. Eight capacitors were placed in an evenly distributed manner through each coil to achieve resonance. A small slab bridge was installed at each crossing point between the coil elements to avoid mixing voxels. In addition to the PA coils, a band-pass filter (BPF) BC coil was included in the simulations as an RF Tx-only coil, as shown in Fig 1(E). BC coil comprised of 16 legs placed around a cylinder, with a radius of 300mm and the height of 140mm. Finally, the combined ||B_1_|| distribution maps were obtained by superimposing individual RF transmission (||B_1_^+^||)-fields of the local Tx-only coil and RF reception (||B_1_^−^||)-fields of the four different PA coils as follows:

$$Combined\;||B1||-field=\sqrt{{\left(||B_{1}^{+}||\right)}^{2}+{\left(||B_{1}^{-}||\right)}^{2}}$$ (1)

The **||**B_1_**||** maps were combined using the sum of squares (SOS) method from **||**B_1_^+^**||** and **||**B_1_^−^**||** fields, which corresponded to the commonly used MR reconstruction in PA coils.

### Construction of local Tx-only birdcage coil and Rx-only PA coil

**Fig 2:**
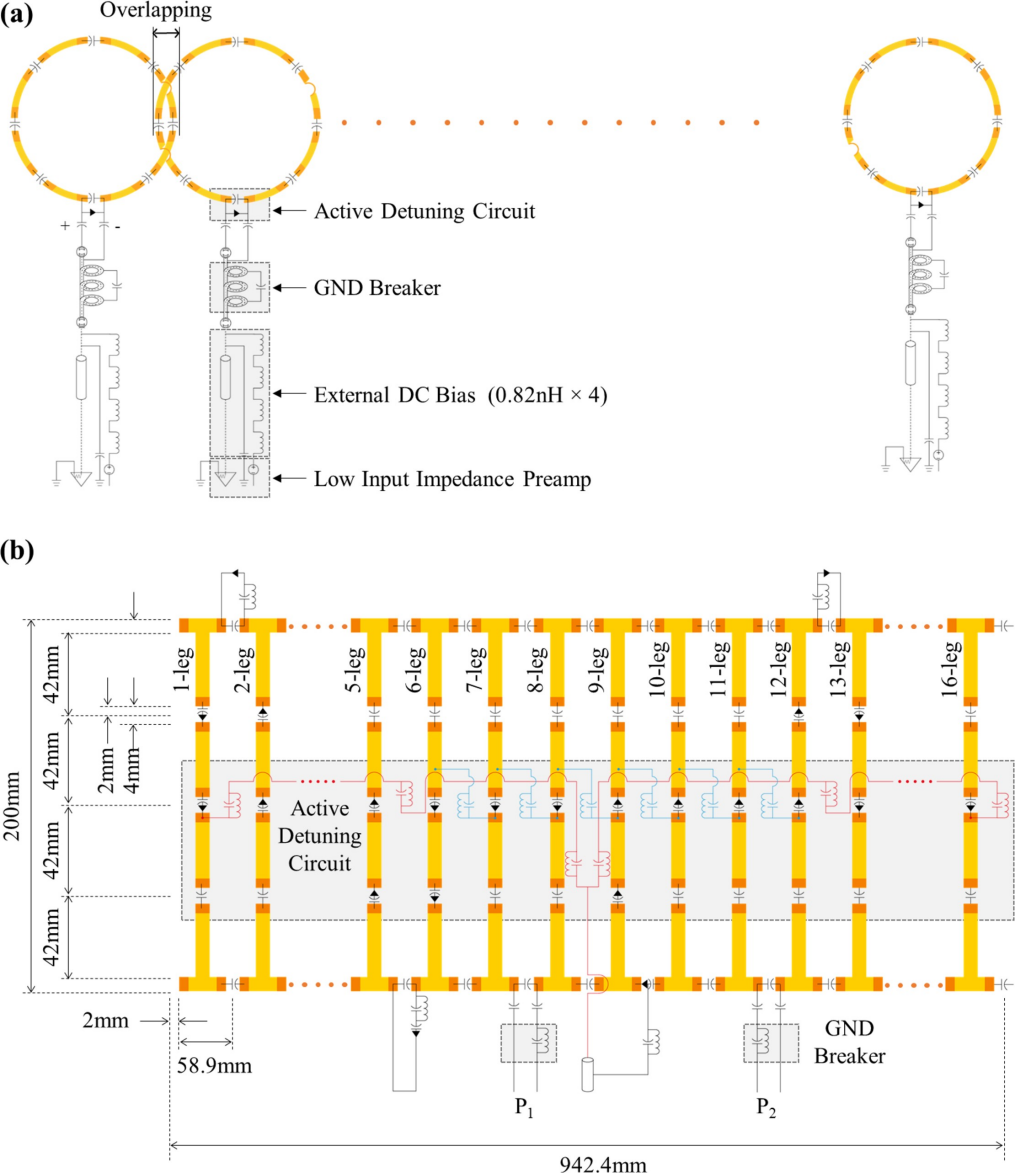
(a) Circuit diagram for overlapping coil elements in the *n*-channel PA coils and (b) circuit diagram of the 16-leg band-pass filter BC coil.

Five coils, namely, a BPF BC coil and four PA coils with 4, 8, 12, and 16 channels, were built according to the models presented in the numerical simulations. The dimensions of the coils were set as described in the previous section. The coils were manufactured using a cylindrical acrylic case as the base and by attaching a flexible polyimide printed circuit board to form the coil elements. For the PA coils, the width of the copper lines was 10mm. The PA coils were cut at eight equidistant locations to attach the tuning capacitors (C_t_). The circuit schematic for the loop coils in the PA coils, wherein two additional gaps are incorporated to include a copper bridge at the crossing point between the overlapping coil elements, is presented in Fig 2(A). An active detuning (AD) circuit, comprising a PIN diode (Micro semiconductor UM9415 diodes), a tuning-matching capacitor (C_tm_), and a matching capacitor (C_m_), was included. During the Rx mode, the PIN diode was turned off, allowing the PA coils to operate in the resonance mode and acquire the MR signal. In contrast, the PIN diode was set on during the RF Tx mode, making the PA coils enter the off-resonance state, thereby avoiding their interaction with the Tx coil at the same time. The coil output was connected to a ground (GND) breaker comprising a semi-rigid coaxial cable (UT70-50; Huber-Suhner, Essex, VT, USA) to function as a band-rejection filter and to the external DC bias circuit for controlling the PIN diode. In this circuitry, four RF chokes (0.82nH) were implemented for blocking the RF Tx when the PIN diode was set on. All the PA coils were connected to a low-noise amplifier (Siemens Medical Solution), with a noise level lower than 0.5dB and a gain of ~26dB. The schematic of the BC coil, with 16 legs connected with end-rings at the top and bottom is shown in Fig 2(B). Three segmented capacitors were employed as BPFs, and an AD circuit was also included. The bench measurements were performed using a calibrated network analyzer (VNA, 8753ES; Agilent Technologies). Each coil was precisely tuned to 300MHz, and the impedance was matched to 50 Ω.

#### Imaging set-up

The MRI experiments were conducted using a 7T scanner (Siemens Medical Solutions, Erlangen, Germany). The images were acquired from a male healthy volunteer of 28 years old. Written informed consent was obtained from the volunteer, and the study was approved by the Institutional Review Boards of Gachon University Gil Medical Center. The brain images were obtained with each of the PA coils under the local Tx-only mode of the BC coil to compare SNR and SNV produced at different R-factors: w/o R-factor (R_1_) and w/ three R-factors (R_2_, R_3_, and R_4_). T2*-weighted gradient echo images were obtained at a repetition time (TR) of 760ms and an echo time (TE) of 17.8ms. The local Tx-only mode of the BC coil was powered by quadrature RF excitation with a flip angle (FA) of 30°; an 8-kW amplifier (LPPA, Dressler, Germany) was used. The MR images were acquired with a matrix size of 896×1024, a FOV of 224×256 mm, and a slice thickness of 2mm. For each PA coil with *n* receiving channels, the final reconstructed MR images were combined using the SOS method from the *n* images obtained with each receiving channel, as follows:

$$I=\sqrt{\sum_{k=1}^{n}\left({Ir}_{k}^{2}+{Ii}_{k}^{2}\right)}$$ (2)

Where, *I* is the combined image, and *I*_*r*_ and *I*_*i*_ are the real and imaginary parts of the images acquired with *n* receivers, respectively. SNR was computed by taking the mean value *(μ*) of a region of interest (ROI) located inside the brain image and by computing the standard deviation (σ) of a selected ROI located in the background. Finally, SNR was calculated using the ratio of *μ*/σ. The images were divided into 20 rectangular cells in the vertical and horizontal directions to compute SNV, where the SNV is defined as the ratio of of *μ*/σ computed at each cell. To investigate PI performance, a GRAPPA algorithm was applied with different R-factors of R_2_, R_3_, and R_4_. The reduction in *k*-space was applied only in the phase-encoding direction. MR images were acquired with reduced *k*-space data for each *n* receiving channel [TR/TE = 760/17.8ms; FA = 30°]. For the brain imaging experiments, all PA coils *w*/ and *w*/*o* R-factors were compared in terms of the SNR and SNV.

## Results

### ||B_1_|| field analysis

**Fig 3:**
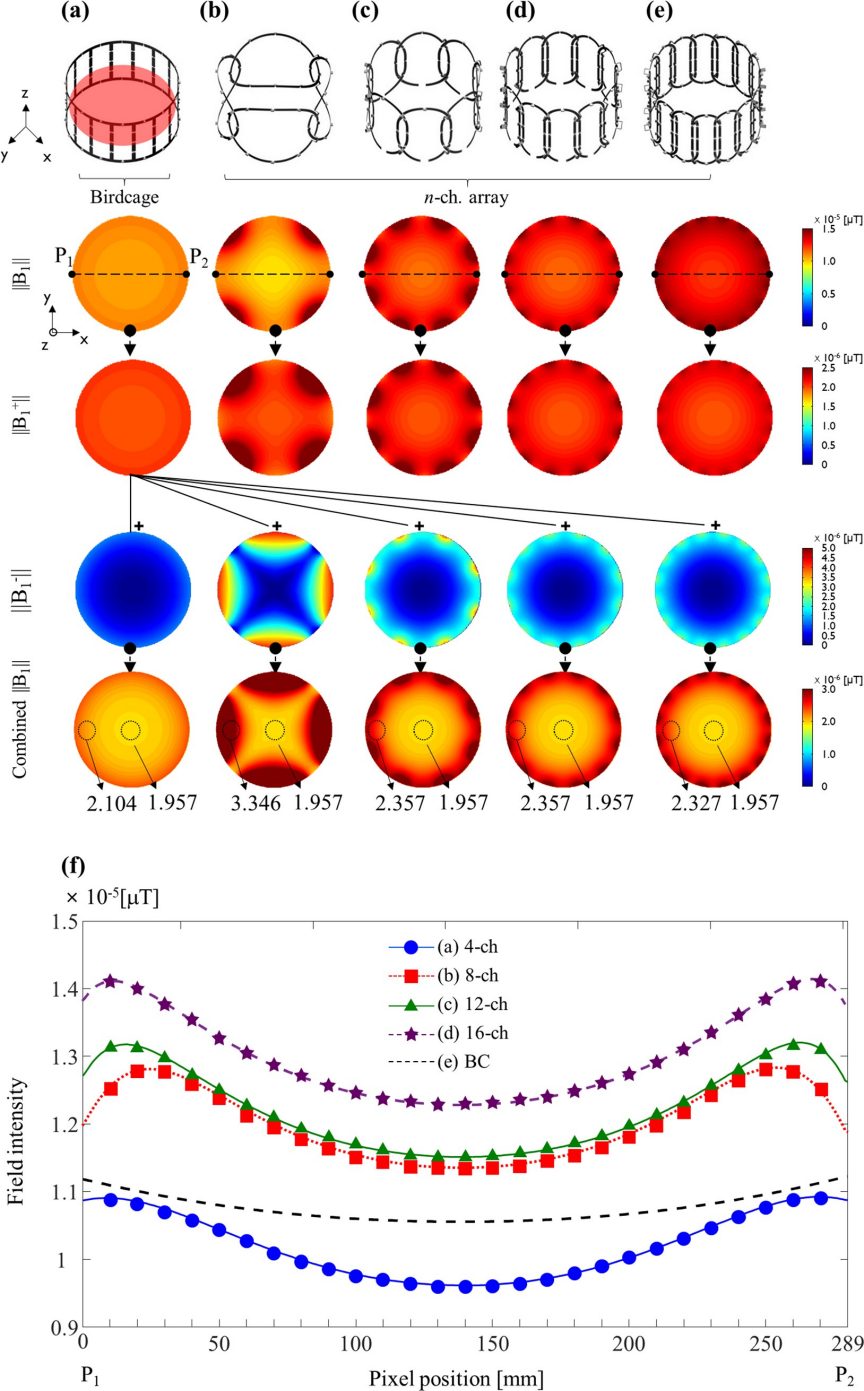
(a–e) Computational calculation of ||B_1_||, ||B_1_^+^||, and ||B_1_^−^|| fields for five coils, including the four PA coils and a BC coil, and the combined ||B_1_|| field. (f) 1D profiles of the five coils in P_1_ to P_2_ of the central axial slice.

**||**B_1_**||**, **||**B_1_^+^**||**, and |**|**B_1_^−^**||** distributions with an oil-based phantom were obtained using a simulation software for the BPF BC coil [Fig 3(A)])] and the PA coils [Fig 3(B)–3(E)]. Higher **||**B_1_**||-**field sensitivity and homogeneity was associated with more number of channels. ||B_1_||-field strength produced by the BPF BC coil showed the lowest sensitivity despite its homogenously distributed |**|**B_1_**||** field. The value of *μ*/σ for the ||B_1_^+^||-field (μT) produced by the BC coil was 0.44 (2.16 /4.88) at the central point of an axial (*xy*)-slice. The *μ*/σ for the PA coils with 4, 8, 12, and 16 channels were 2.46 (3.43/1.39), 2.42 (2.60/1.07), 2.54 (2.54/1.00), and 2.54 (2.57/1.01), respectively. The ||B_1_^+^||-field produced under Tx-only mode of the BC coil and ||B_1_^−^||-fields produced by the *n* channel PA coils were combined using SOS methods [Fig 3, last row]. In terms of the homogeneity of the combined ||B_1_||-field map, the PA coil with 4 receiver channels showed lower field variation at the center of the imaging area, while maintaining high field intensity at the periphery of the coil. In particular, the 16-channel PA coil exhibited lower field intensity at the periphery than the other PA coils, but it showed a more homogeneous ||B_1_||-field. This was expected since ||B_1_||-field homogeneity is proportional to the area of the coil.

#### Coil building

**Fig 4:**
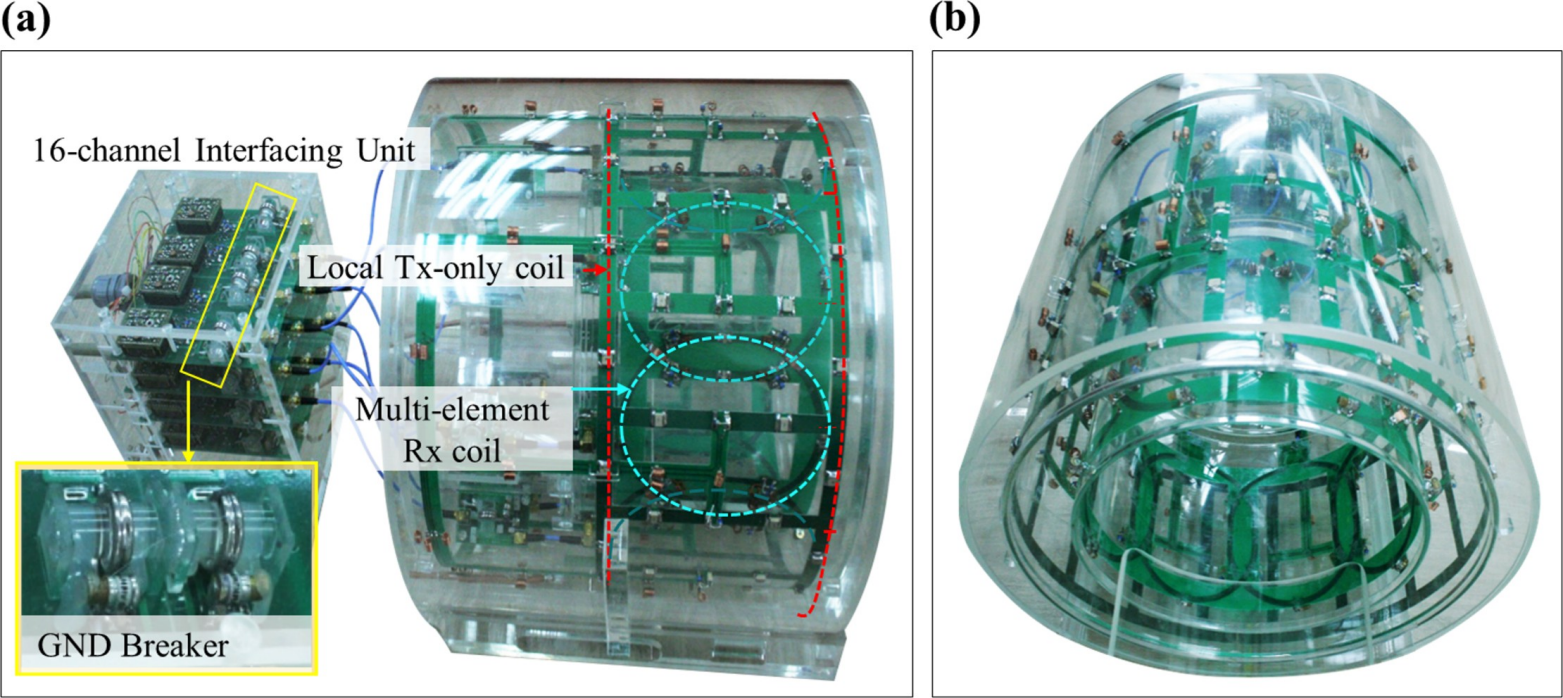
(a) Side view of images obtained under the Tx-only mode of the BC coil and PA coils, including the interfacing unit, and (b) front view of combined two coils.

The BC coil along with the *n*-channel PA coil is presented in Fig 4. The *n*-channel interfacing unit comprised GND breakers and low-noise preamplifiers for each PA coil element, which were placed in the rear part. The positions of the coil elements in the PA coils are indicated by white dashed lines, and the length of the BC coil is shown as a red dotted line. The interfacing unit was built to accommodate a maximum of 16 receiver channels. The *n*-channel PA coils were decoupled using overlapping geometries.

**Fig 5:**
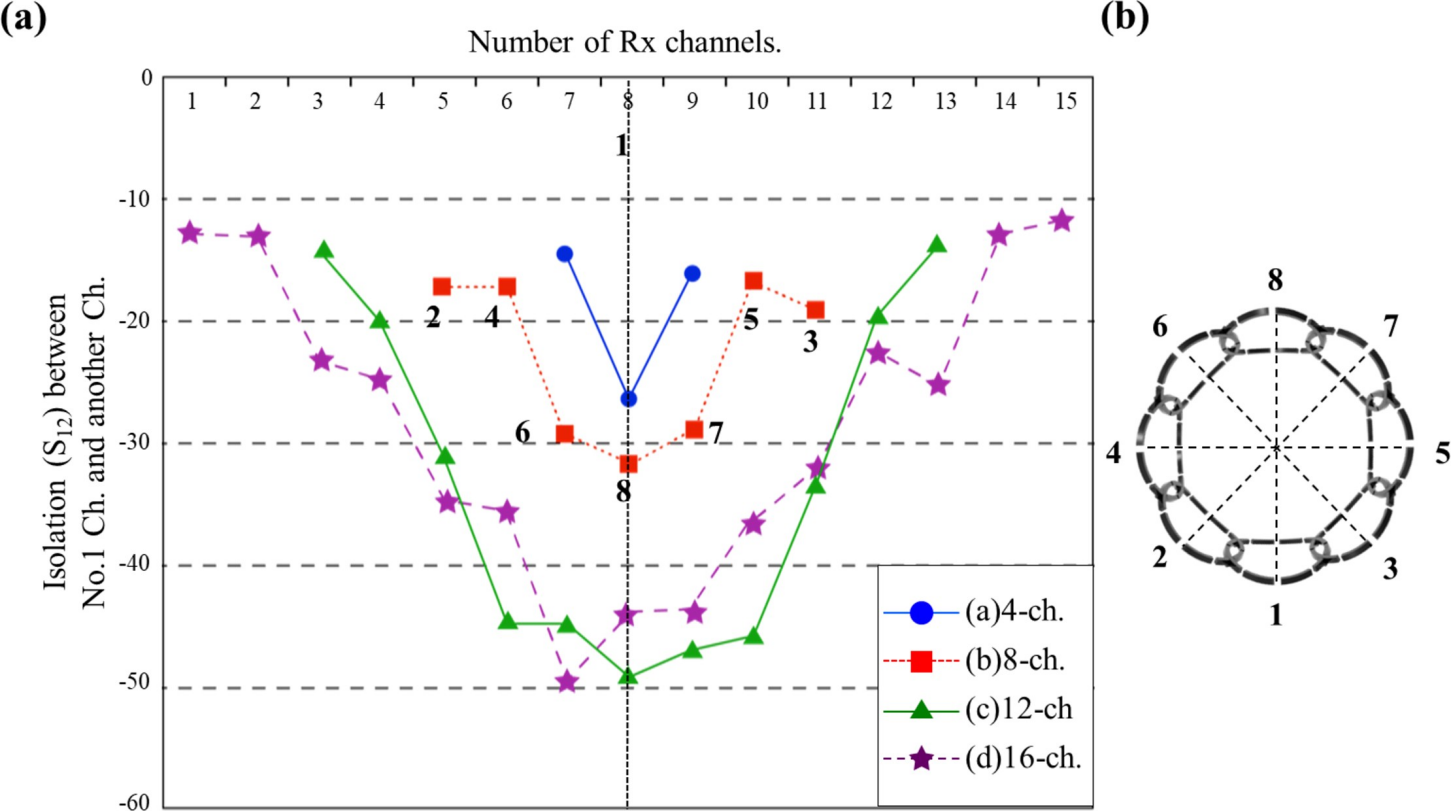
(a) Decoupling levels between coil elements using channel 1 as the reference and (b) diagram to reference the coil positions.

Isolation of the individual coil elements was achieved by measuring the transmission characteristic (S_21_) parameters. The plots of the isolation levels among channels against the number of channels are shown in Fig 5(A), and the labeling of the channels is shown in Fig 5(B). The 4-channel PA coils showed isolation below −20dB with adjacent coils when unloaded, and the isolation was lower than −14.8 dB with the adjacent coils and −26.2dB with the coil on the opposite direction when loaded. Similarly, the isolation values for the 8-, 12-, and 16-channel PA coils with the adjacent channels were respectively −16.8dB, −13.7dB, and −11.7dB. The maximum isolation was acquired with the farthest coil on the opposite side, with S_21_ values of −31.7dB, −49dB, and −49.5dB for the 8-, 12-, and 16-channel PA coils, respectively. The mutual coupling upon changing the reference coil produced similar isolation values. The isolation obtained with the 12- and 16-channel PA coils clearly showed a greater improvement compared with that obtained with the 8- and 4-channel PA coils. However, the isolation obtained with the 16-channel PA coil improved by only 1% compared with that obtained with the 12-channel PA coil. The higher isolation achieved with the 12- and 16-channel PA coils can be related to the area of each coil element since there is a lower level of induction of the propagation field into coils that are located farther from the diameter. Further, for the 16-channel PA coil, the two adjacent coils showed the lowest decoupling of approximately −12dB. However, significant isolation was achieved after the third channel. The increase in the isolation between the first two channels and the third channel was 47%.

#### SNR distribution for the four PA coils

**Fig 6:**
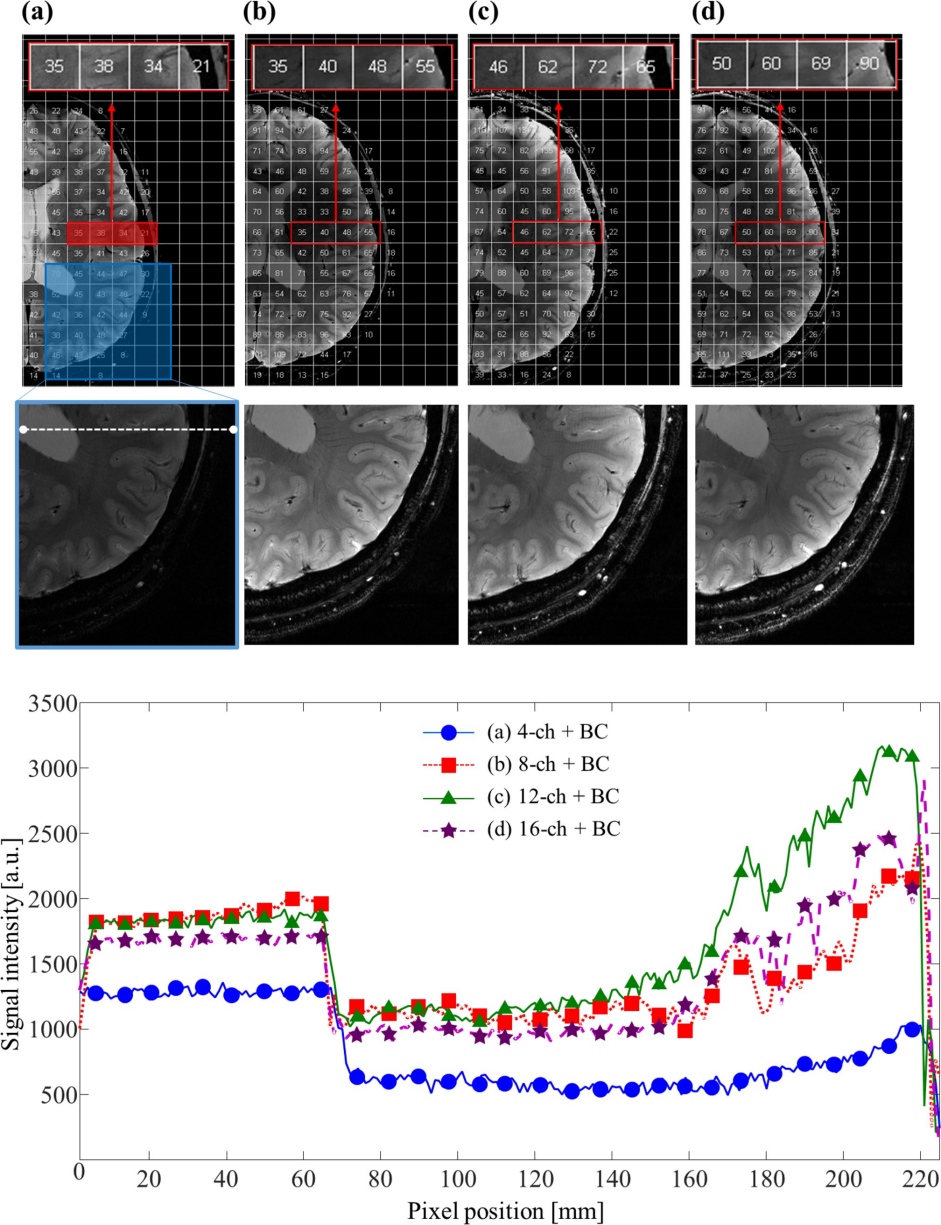
(a–e) MR images obtained with 4-, 8-, 12-, and 16-channel PA coils under R_1_. (e) Line profile of P_3_ to P_4_ comparing each PA coil under the Tx-only mode of the BC coil.

To evaluate the performance of the PA coils in obtaining MR images, first, T_2_*-weighted images of the human brain were under R_1_ condition. The MR images acquired with each PA coils are shown in Fig 6(A)–6(D). The images were reconstructed and combined using the rooted SOS methods. Each PA coil exhibited a high SNV for the entire brain although there was a significant reduction signal intensity (SI) into the interior of the brain compared with that at the periphery, except for the 4-channel PA coil because the signal penetration is proportional to the radius of the single-coil element [15]. In Fig 6(A)–6(D), the highlighted four cells show the computed SNV depending on the PA coil used. The *μ* values of these four cells were 35.6, 41.0, 60.0, and 59.7 for the each PA coils, respectively. SIs for the 12- and 16-channel PA coils were similar, which might be because of the short distance and low isolation between the adjacent coils, as discussed earlier. SI profiles were obtained from the center of the brain along the P_3_ to P_4_ position (Fig 6, bottom panels). SI profile for the 4-channel PA coil showed higher signal homogeneity, despite a lower SI value compared with the other coils. The σ values normalized by the *μ* from the plot at the center region were 12.52%, 13.88%, 16.13%, and 12% for the four PA coils, respectively.

#### Spatial noise distribution for parallel imaging capability

**Fig 7:**
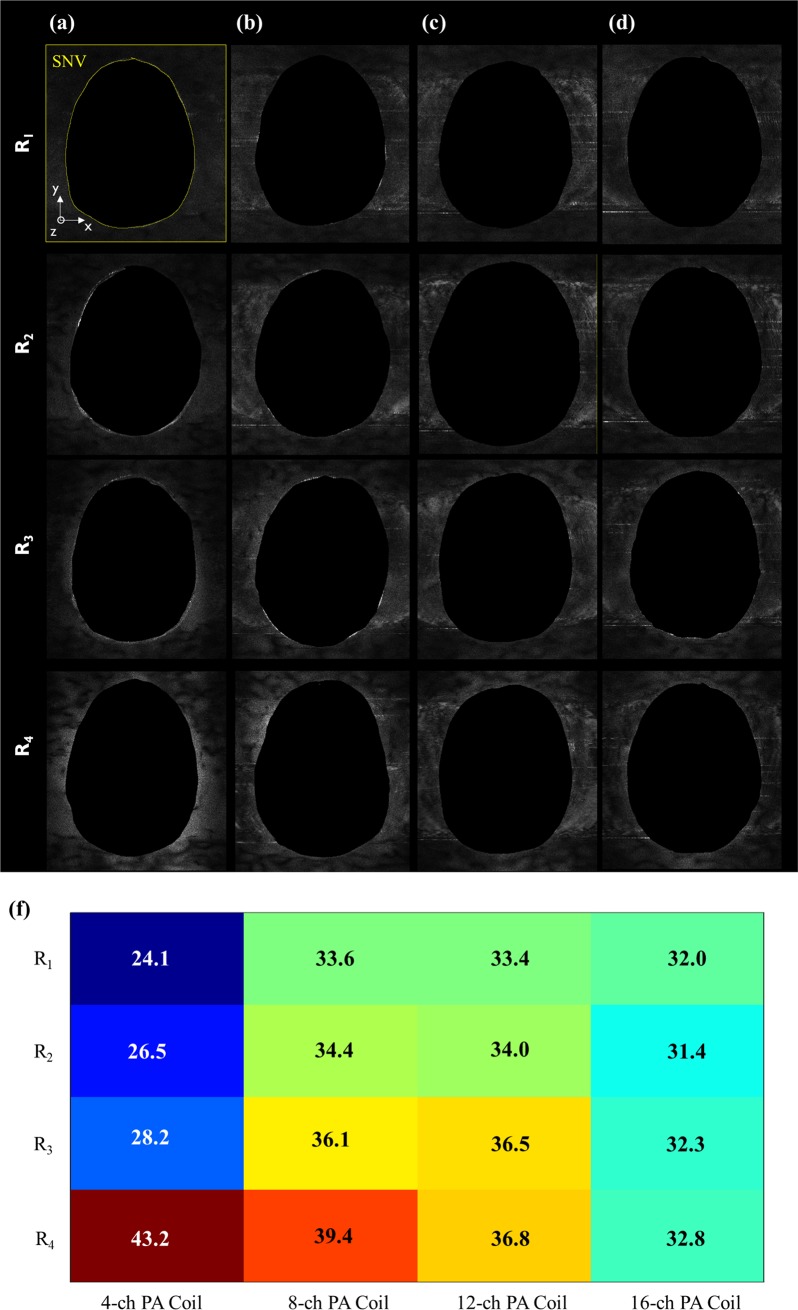
Spatial noise distribution for images reconstructed using the GRAPPA algorithm with different acceleration factors of *w*/ (R_1_) and *w*/ (R_2,_ R_3_, R_4_) and (f) the value of noise was measured for four PA coils.

The MR images were acquired under R_2_, R_3_, and R_4_ conditions and reconstructed using the GRAPPA algorithm with 70 acquisition lines as reference. The acquisition time for a fully sampled image was 12min 48s; this time was reduced to 6min 51s, 4min 53s, and 3min 53s under R_2_, R_3_, and R_4_, respectively. The images reconstructed with a reduction factor of 4 for the corresponding coils along with σ values are shown in Fig 7. SNV distributions with the corresponding acceleration factor for each PA coil. The 16-channel PA coils showed lower SNV at higher R-factors compared with the coils with fewer elements. The reference images were set for reconstruction *w/o* R-factor. Under R_2_ and R_3_ conditions, the 4-channel PA coil showed reduced SNR compared with the other PA coils; the 16-channel PA coil was the best SNR under R_2_ and R_3_. Finally, under R_4_, the 16-channel PA coil showed an σ of 27.02, indicating an improvement compared with the performances of the other coils. The performance of each coil is expressed as the σ values with different R-factors.

## Discussion and conclusion

In this study, four configurations of PA coils were designed and built, comprising of 4, 8, 12, and 16 channels. EM simulations were performed to obtain ||B_1_||-field distributions for each coil. A more uniform ||B_1_||-field could be achieved in the periphery when higher number of channels was used. However, the penetration depth of the field decreased with the increase in the number of coil elements. Increasing the number of channels increases SNR at the periphery compared with that in the interior of the brain. The coils were built using overlapping geometry to reduce the inductance coupling. The level of isolation between the coils averaged −20dB. The isolation level was low (approximately −10dB) for the 16-channel coil only. The simulation results were validated by performing *in vivo* brain imaging with each coil. High SNV levels were obtained at the periphery of the brain. Notably, the SI was more uniform at the center of the FOV when the 4-channel PA coil was used compared with that when the other PA coils were used. In addition, SI was apparently truncated for 8-, 12-, and 16-channel PA coils. The comparison of the performance of each coil for in terms of PI revealed that the 12- and 16-channel PA coils produced high SNR levels under R_4_. Coil selection for neuroimaging should be performed with caution because different numbers of Rx channels in PA coils produce different SNR and SNV. The use of a collection of smaller surface coils can contribute to high RF signal sensitivity in terms of the anatomical coverage of the brain and can facilitate PI. In addition, our research can be extended to other types of array coils. The optimization is possible because fewer channels with larger elements can provide a more uniform SI but a poorer PI ability for the same object coverage, whereas more but smaller coils can facilitate PI at higher acceleration factors and can produce high SNR at the periphery of the imaging object.
